# Insights Into Giant Intrapulmonary Teratomas in Infants: A Case Report and Literature Review

**DOI:** 10.7759/cureus.62937

**Published:** 2024-06-22

**Authors:** Sara E Marhoon, Ali H Ali, Osama M Abdelmoneim, Tarek Eldesoky

**Affiliations:** 1 College of Medicine, Mansoura University, Mansoura, EGY; 2 Pediatrics, Mansoura University Children Hospital, Mansoura, EGY

**Keywords:** case report, benign mature cystic teratoma, giant, infancy, thoracotomy, intrapulmonary teratoma

## Abstract

Mature cystic teratomas exhibit a variety of tissues within their pathology. In adults, teratomas typically originate in the gonads. However, one of the rarest origins is the lung, making intrapulmonary teratoma (IPT) exceedingly uncommon. In infants, extragonadal teratomas are more common, with only two cases of IPT reported in the literature. While the clinical presentation in infants and adults is similar, fever appears to be unique to infant cases. We present a case of a one-year-old female who exhibited respiratory distress and fever. A chest X-ray revealed an opaque right hemithorax, initially leading to a diagnosis of pneumonia. Despite intravenous (IV) antibiotic treatment, there was no improvement. A subsequent chest computed tomography (CT) scan revealed a large mass with heterogeneous densities occupying the entire right hemithorax, indicative of IPT. The mass was successfully excised, and the infant was discharged on the 11th postoperative day without complications.

This case adds to the limited literature on giant IPT in infants compared to the two previously published cases.

## Introduction

Mature cystic teratomas are benign cell tumors mostly located in the gonads [1]. It is rare to have extragonadal germ cell tumors, but when this occurs, the most common site is the mediastinum [2]. However, other rare sites include the retroperitoneum, sacrococcygeal area, and, more rarely, the lung, in which case it is considered an intrapulmonary teratoma (IPT) [3]. In contrast, the primary site in neonates and young children is extragonadal, particularly the sacrococcygeal region [4]. The incidence of teratomas follows a bimodal pattern: the first peak occurs between birth and four years of age, and the second peak often starts at puberty and continues until the fourth to fifth decades of life [5,6].

The clinical presentation of patients with IPT is nonspecific and includes chest pain, persistent cough, and hemoptysis, making them indistinguishable from other diseases [7]. The initial diagnostic tool is a chest X-ray. Subsequently, computed tomography (CT) is used for further evaluation of the nature, location, and relationship of the tumor to the surrounding structures. Magnetic resonance imaging (MRI) provides superior results compared to CT in detecting the extent of the spread [8].

Herein, we present a comprehensive case study detailing the journey of an infant with IPT, from the initial presentation to a successful complete cure.

## Case presentation

A one-year-old female infant presented to our hospital with low-grade fever, dry cough, and dyspnea persisting for three days. She was born at term via cesarean section with an unremarkable past medical and surgical history.

Upon clinical evaluation, the infant weighed 7.5 kilograms (kg). Vital signs showed a pulse count of 130 beats/min, blood pressure of 100/60 mmHg, temperature of 37 °C, and a respiratory rate of 42 breaths/min, indicating respiratory distress. Auscultation revealed diminished air entry on the right side.

Routine investigations showed mild leukocytosis, normocytic hypochromic anemia, and mildly elevated C-reactive protein (CRP) (Table 1).

**Table 1:** Initial laboratory investigations.

Test	Result	Reference range
Complete blood count		
White blood cell count	12.38 k/uL	4.1-10.9 k/uL
Red blood cell count	4.571 m/uL	4.20-6.30 m/uL
Hemoglobin	11.17 g/dL	12-18 g/dL
Mean corpuscular volume	81.67 fL	80-97 fL
Mean corpuscular hemoglobin	24.43 pg	26-32 pg
Mean corpuscular hemoglobin concentration	29.91 g/dL	31-36 g/dL
Platelets	362 k/uL	140-440 K/μL
Inflammatory markers		
C-reactive protein	20 mg/L	Up to 10 mg/L

**Figure 1:**
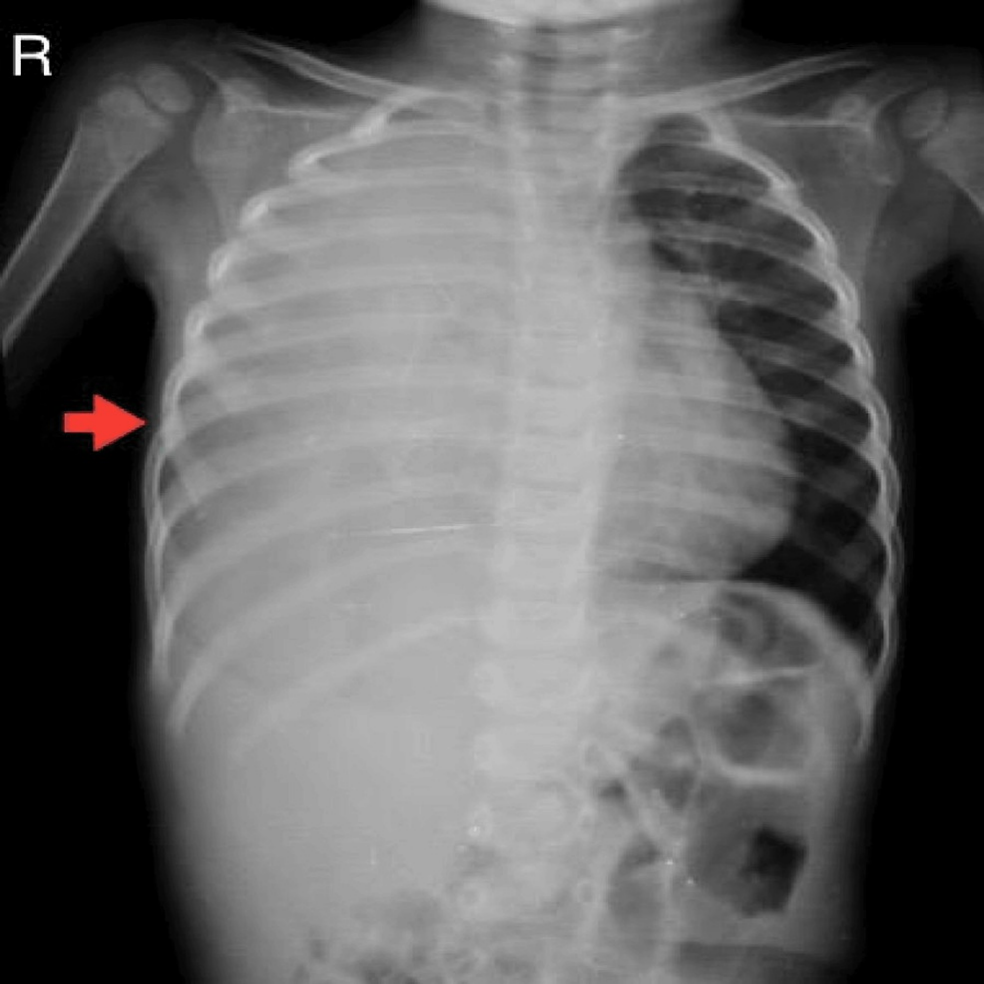
A representative chest X-ray shows an opaque right hemithorax with a mediastinal shift to the opposite side.

A chest X-ray revealed a right-sided opaque hemithorax with a mediastinal shift to the left side (Figure 1). A chest ultrasound (US) examination confirmed the presence of moderate right-sided pleural effusion with underlying septations. Consequently, a chest tube was inserted, extracting clear, yellowish pleural fluid with a glucose content of 62 mg/dL, devoid of cells and organisms. Upon the initial diagnosis of pneumonia, intravenous (IV) antibiotics were administered, but there was no evident improvement.

**Figure 2:**
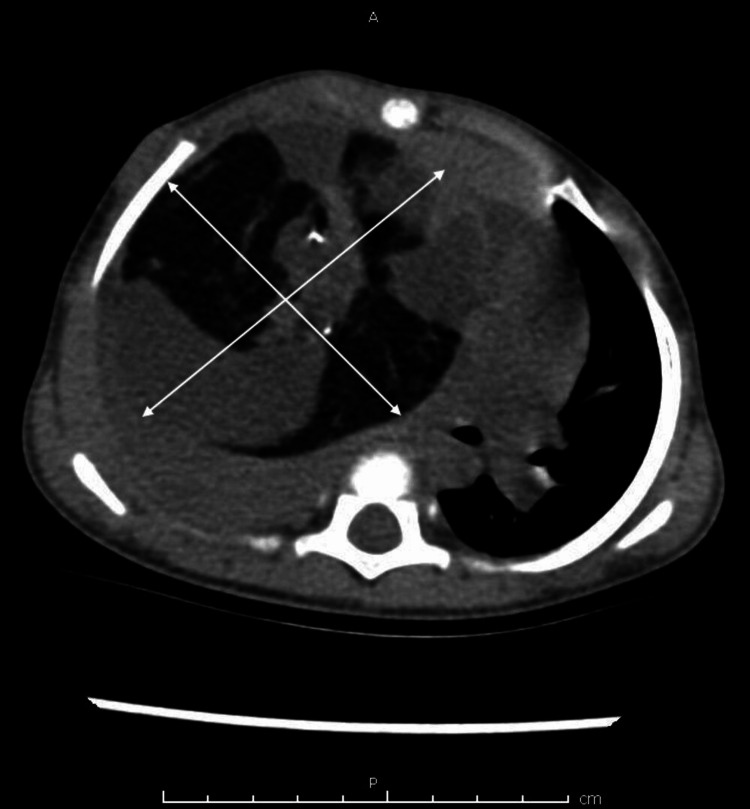
An NCCT chest revealing a mass measuring 10x8 cm that occupied the entire right side of the chest. The mass exhibited a heterogeneous composition, characterized by mixed fat, cystic, and calcification densities with moderate non-homogeneous enhancement. The mass caused displacement of the mediastinum to the opposite side and a complete collapse of the underlying lung. NCCT: non-contrast computed tomography

To further clarify the case, a non-contrast computed tomography (NCCT) chest scan was performed, revealing a giant mass measuring 10x8 cm that occupied the entire right side of the chest (Figure 2). The mass exhibited a heterogeneous composition, characterized by mixed fat, cystic, and calcification densities with moderate non-homogeneous enhancement occupying the entire right lung. Notably, it exerted compression on the mediastinum, causing its displacement to the opposite side, and resulted in a complete collapse of the underlying lung, indicative of a possible IPT.

Three months later, a scheduled right-sided thoracotomy was performed, successfully excising the teratoma. Postoperatively, the infant was transferred to the pediatric intensive care unit (PICU) while intubated. To minimize the risk of postoperative complications, chest physiotherapy was initiated on the day of surgery.

Three specimens were analyzed pathologically. The first two consisted of the mass cut into two halves, measuring 10x8.5x3.5 cm and 12x8x4.5 cm, respectively. Upon dissection, they exhibited soft, fatty, tan-whitish cut sections with bony parts, along with multiple cystic spaces filled with mucinous clear fluid oozing from these spaces. Additionally, the third specimen consisted of 60 ml of yellowish fluid.

Microscopic examination revealed the presence of multiple cystic structures, some lined by stratified squamous epithelium, while others were lined by columnar epithelium with goblet cells. Additionally, wider areas of mature brain tissue, fat, and nerves were observed alongside a background of thymic tissue. No immature tissue was detected. Furthermore, the aspirated fluid was found to be acellular, confirming the final diagnosis of a mature cystic teratoma (Stage 0).

**Figure 3:**
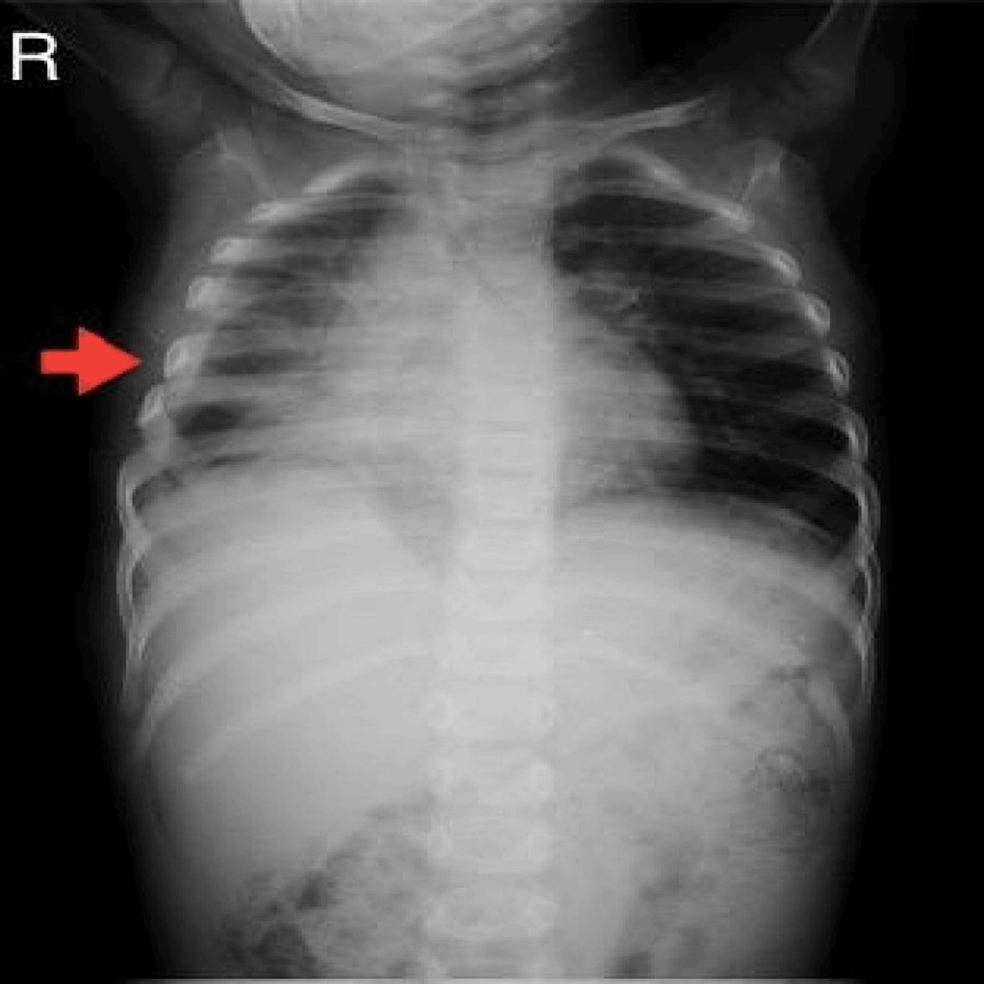
A follow-up plain chest X-ray confirmed the lung re-expansion.

On the ninth postoperative day, lung expansion was confirmed via a chest X-ray (Figure 3). The patient was discharged on the 11th day postoperative day, with scheduled outpatient clinic appointments to ensure thorough follow-up care.

## Discussion

Mature cystic teratomas are the most common type of germ cell tumors [1], typically originating in the gonads but occasionally found in various anatomical sites such as the sacrococcygeal region and the mediastinum, in order of decreasing frequencies [9]. Intrathoracic teratomas primarily arise within the mediastinum [10], making IPT an exceedingly rare entity since it was first reported by Mohr in 1839 [1,11]. Furthermore, IPT is predominantly diagnosed in adulthood [6], rendering its occurrence in infancy exceptionally uncommon, particularly when accompanied by a substantial size encompassing the entire lung, as observed in this case.

Schlumberger's theory, proposed in 1946, suggests that IPT may originate from the thymus as a derivative of the third pharyngeal pouch [9]. This might be supported by the predominance of the tumor in the upper lung lobes (65%) on both sides [10,12]. The presence of thymic tissue in our specimen further bolsters this theory. Notably, our case exhibited a distinct feature of a wide area of mature brain tissue within the teratoma, adding to the complexity of its composition.

Estevez, in his review of the latest IPT cases reported since 2010, found a mean age of 25.5 years, with the majority of cases occurring in the third decade of life and only one infantile case [1]. To our knowledge, only two IPT cases in infants have been reported to date, making our case the third documented instance [6,9]. These findings are summarized and compared in Table 2.

**Table 2:** Summary of the cases of IPT in infancy. IPT: intrapulmonary teratoma

	Sex	Age	Symptoms	Lung side	Lung lobe	Size (cm)	Operation	Pathology
Our Case	Female	1 year	Low-grade fever, cough, and dyspnea	Right	All lobes	10x8	Excision	Stratified squamous epithelium and columnar epithelium with goblet cells, brain tissue, fat, nerves, and thymic tissue.
[6]	Male	6 months	Fever	Left	Upper lobe	8.3x4.7	Excision	Stratified squamous epithelium, respiratory epithelium, mature adipose tissue, cartilage, gastro-intestinal and pancreatic tissue elements.
[9]	Female	11 months	Low-grade fever, cough, and dyspnea	Right	All lobes	7.3x11.2x8.5	Excision	Cartilage and bony structures.

IPT commonly manifests with symptoms such as cough, dyspnea, and chest pain [1]. A notable but rare symptom, trichoptysis, characterized by coughing and expectoration of hair, results from communication between the teratoma and the main bronchus [11,13]. Fever emerged as a cardinal symptom in our case and all reported infant cases, although it is less frequently observed in other age groups. Together with cough and dyspnea, fever led to the initial presumptive diagnosis of pneumonia in our case and a similar case report [9].

IPT typically has a diameter of around 3 cm [11]. The discovery of a giant teratoma occupying the entire lung, as seen in infant cases including ours, is an extraordinary finding that might be unique to the infant age group [6,9].

Diagnosing IPT preoperatively poses a challenge due to its vague presentation. Therefore, a comprehensive radiological assessment is essential. This typically begins with a standard chest X-ray, followed by a CT chest to evaluate opacity and differentiate various tissue densities [10]. In our case, the CT chest revealed the characteristic heterogeneity of teratomas, including mixed fat, cystic, and calcification densities, as well as complete lung collapse due to obstruction by the giant teratoma. While MRI can aid in surgical planning by delineating anatomical relations to mediastinal structures, it was unnecessary in our case [6].

Complete surgical excision is the treatment of choice for IPT, necessitating timely intervention to avoid potential complications. These complications include bronchial obstruction, pleural effusion, atelectasis, pneumonia, and rupture [6,14]. Therefore, prompt and thorough surgical removal is essential to ensure optimal patient outcomes and minimize risks associated with IPT.

## Conclusions

IPT is rare in infants. Due to its nonspecific clinical presentations, radiology is essential for differentiating teratomas from other potential thoracic masses, as it reveals characteristic heterogeneous tissue densities. Complete surgical resection is crucial to prevent complications. Further studies are needed to illuminate the key differences between IPT in infancy and adulthood, possible associated genetic mutations, and long-term outcomes after complete excision.
